# Overweight/obesity accelerates proteinuria progression in type 2 diabetes post-COVID-19: a comparison with a pre-pandemic uninfected cohort

**DOI:** 10.3389/fendo.2026.1804593

**Published:** 2026-04-17

**Authors:** Jiajun Wu, Liping Gu, Qin Zhen, Jiaying Yang, Yizhe Wang, Yongcheng Zhang, Yizhen Chen, Fang Liu, Xiaoying Ding, Yufan Wang, Na Li

**Affiliations:** 1 Department of Endocrinology and Metabolism, Shanghai General Hospital, Shanghai Jiao Tong University School of Medicine, Shanghai, China; 2 University of Shanghai for Science and Technology, Shanghai, China

**Keywords:** COVID-19, overweight/obesity, proteinuria progression, retrospective study, type 2 diabetes mellitus

## Abstract

**Aims:**

To assess whether overweight/obesity accelerates proteinuria progression in patients with type 2 diabetes mellitus (T2DM) after coronavirus disease 2019 (COVID-19) infection.

**Methods:**

This retrospective study included 688 patients with T2DM confirmed to have COVID-19 infection and 502 pre-pandemic uninfected patients with T2DM for comparative analysis. Participants were categorized based on body mass index (BMI) into normal weight (BMI <24 kg/m^2^) and overweight/obese (BMI ≥24 kg/m^2^) groups. Proteinuria progression, defined as an increase in albuminuria stage, was assessed within three months pre- and post-infection. Multiple logistic regression analysis was used to evaluate the association between body weight status and proteinuria progression after COVID-19 infection.

**Results:**

Of the 688 infected participants, 8.5% experienced proteinuria progression. Within the infected cohort, the incidence of progression in the overweight/obese group (10.8%) was significantly higher than in the normal weight group (4.9%, P = 0.012). Critically, the incidence of progression in the infected overweight/obese group (10.8%) was also significantly higher than that in their uninfected overweight/obese counterparts (5.8%, P = 0.016). In the COVID-19 infection cohort, overweight/obese was associated with a 2.682-fold higher risk of proteinuria progression after COVID-19 infection compared to the normal weight group (OR, 2.682; 95%CI, 1.273-5.648; P = 0.009). Furthermore, every 1 kg/m^2^ increase in BMI was associated with a 13.1% increased risk of proteinuria progression after COVID-19 infection (OR, 1.131; 95% CI, 1.039 -1.23; P = 0.004).

**Conclusions:**

Overweight/obese accelerates proteinuria progression in patients with T2DM following COVID-19 infection. Therefore, emphasizing the importance of obesity management is crucial to prevent renal complications in patients with T2DM after COVID-19.

## 1 Introduction

Severe acute respiratory syndrome coronavirus 2 (SARS-CoV-2) is a highly contagious and pathogenic novel coronavirus that emerged in late 2019, causing the coronavirus disease 2019 (COVID-19) pandemic (1). Since December 2019, SARS-CoV-2 has spread massively, causing more than 700 million infections and over 7 million deaths globally (2). While COVID-19 primarily manifests as an acute respiratory illness, it is known to affect multiple organs, including the kidney (3). The virus exhibits renal tropism, directly infecting renal cells via angiotensin-converting enzyme 2 (ACE2) binding, leading to various forms of kidney injury, which often present clinically as proteinuria. Indeed, early studies on hospitalized COVID-19 patients reported high incidences of renal involvement (75.4%) and proteinuria (65.8%) (4). Furthermore, a cohort study demonstrated that COVID-19, especially in hospitalized cases, is associated with a greater accelerated decline in kidney function (5).

As SARS-CoV-2 infection has become highly prevalent and recurrent globally, understanding modifiable factors that may contribute to post-infection renal decline is an important clinical consideration. Notably, type 2 diabetes mellitus (T2DM) and overweight/obesity represent two of the most significant and rapidly increasing global public health challenges, serving as key risk factors for the development and progression of proteinuria and ultimately end-stage renal disease (ESRD) in T2DM (6). Obesity contributes to renal damage through mechanisms such as the release of pro-inflammatory adipokines and activation of the Renin-Angiotensin-Aldosterone System (RAAS), leading to glomerular ultrafiltration and subsequent proteinuria. The synergistic effect of these comorbidities and COVID-19 infection places this population at an especially high risk for sustained or progressive kidney complications.

Despite the established individual impacts of T2DM, obesity, and COVID-19 on renal health, it remains largely unexplored whether overweight or obesity accelerates the progression of proteinuria in COVID-19-infected patients with T2DM. Therefore, this retrospective study aimed to explore the association between body weight status and proteinuria progression in patients with T2DM before and during the COVID-19 pandemic.

## 2 Materials and methods

### 2.1 Participants

**Figure 1:**
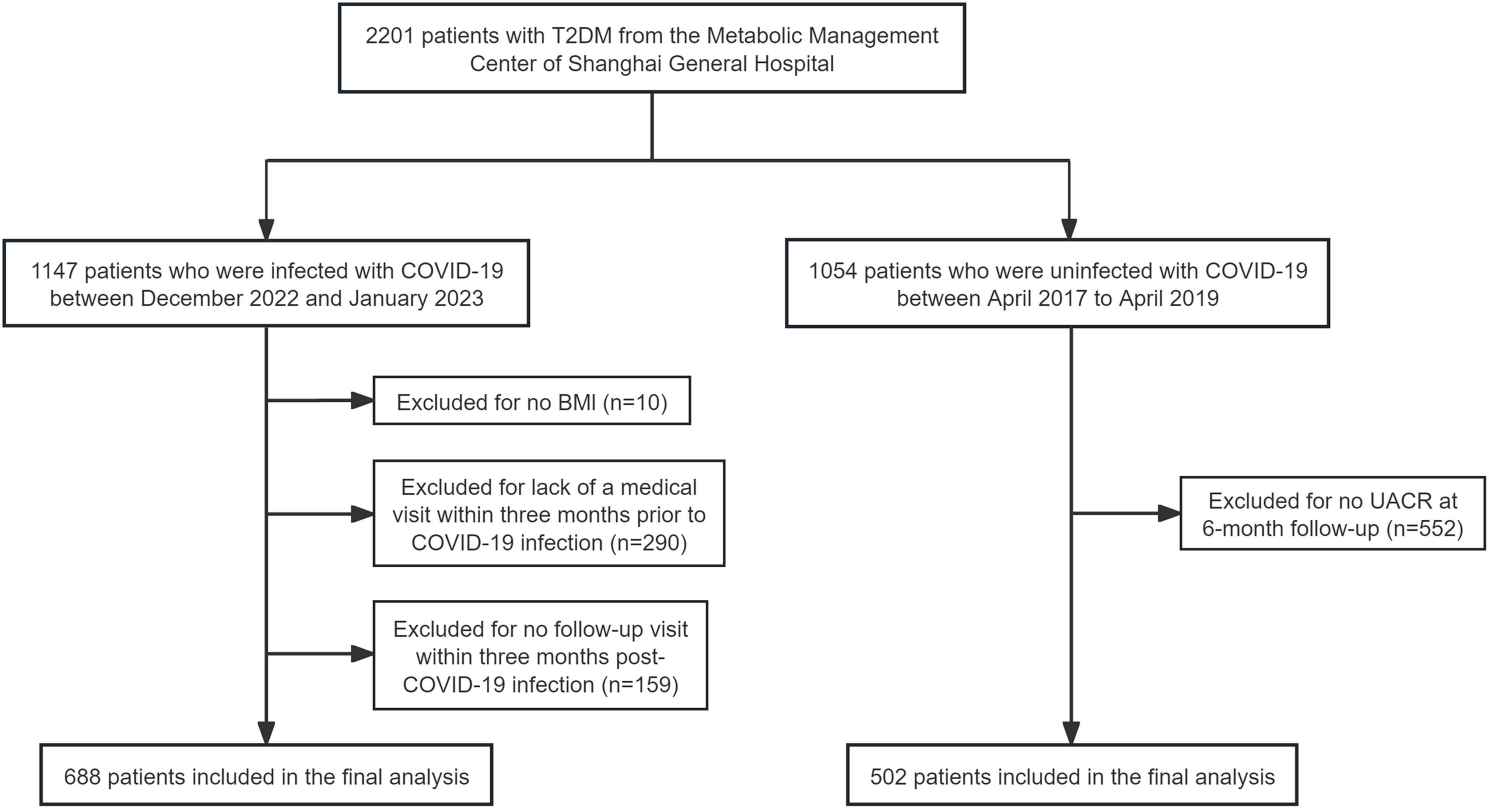
Flow chart of the study participants. A total of 2201 patients with T2DM were recruited from the Metabolic Management Center of Shanghai General Hospital. 1147 patients constituted the COVID-19 cohort, enrolled following a confirmed SARS-CoV-2 infection between December 2022 and January 2023. The remaining 1054 patients comprised the non-COVID-19 cohort, who were enrolled during the pre-pandemic period between April 2017 and April 2019. Of the 1147 patients with COVID-19, 459 were excluded for the following reasons: absence of BMI data (n=10), lack of a medical visit within three months prior to COVID-19 infection (n=290) and no follow-up visit within three months post-COVID-19 infection (n=159). Of the 1054 patients without COVID-19, 552 were excluded due to missing UACR data at 6-month follow-up. Consequently, a total of 688 COVID-19 patients and 502 non-COVID-19 patients were included in the final analysis.

A retrospective cohort study was conducted to investigate the effect of body weight status on proteinuria progression in patients with T2DM after COVID-19 infection. We initially recruited 1147 patients with T2DM who were infected with COVID-19 between December 2022 and January 2023 from the Metabolic Management Center (MMC) of Shanghai General Hospital. All patients were confirmed to have SARS-CoV-2 infection via a positive PCR/antigen nasopharyngeal test. Of the 1147 patients, 459 were excluded for the following reasons: absence of body mass index (BMI) data (n=10), lack of a medical visit within three months prior to COVID-19 infection (n=290), no follow-up visit within three months post-COVID-19 infection (n=159). Consequently, a total of 688 patients were included in the final analysis of the infected cohort (**Figure 1**).

For comparative analysis of the impact of COVID-19 on proteinuria progression, patients with T2DM without COVID-19 infection were also recruited. The detailed study design has been published previously (7). In brief, a total of 502 participants with baseline and 6-month UACR measurements were enrolled from the MMC of Shanghai General Hospital (**Figure 1**). This cohort was recruited between April 2017 to April 2019, a period specifically chosen to exclude COVID-19 infection, as there were no COVID-19 cases in China before November 2019. Although a contemporaneous non-infected cohort was initially considered, the sample size was limited to 134. Therefore, the pre-pandemic cohort was ultimately selected as the primary comparator due to its larger sample size, which was essential to provide adequate statistical power for subgroup analysis stratified by BMI.

This study was approved by the Ethics Committee of Shanghai General Hospital (2017KY209-5C24-1). Written informed consent was obtained from all participants prior to the study. The study was conducted in accordance with the Declaration of Helsinki.

### 2.2 Definitions and measurements

We collected demographic data and medical history including age, sex, anthropometric measurements [body weight (BW), height, body mass index (BMI), waist circumference (WC), hip circumference (HC), waist/hip ratio (WHR)], blood pressure (BP), diabetes duration, smoking history, medication use, and COVID-19 vaccination status. Hemoglobin A1c (HbA1c), urine albumin, urine creatinine and other clinical indexes of glucose and lipid metabolism were recorded within three months pre- and post-COVID-19 infection.

All blood samples were collected following an overnight fasting period (8–10 hours). HbA1c levels were determined using a Lifotronic H8 autoanalyzer (Japan), while plasma insulin was quantified via the Abbott i2000 chemiluminescence systems (United States). Furthermore, a comprehensive biochemical profile encompassing fasting glucose, lipid indices [total cholesterol (TC), triglycerides (TGs), high-density lipoprotein cholesterol (HDL-C) and low-density lipoprotein cholesterol (LDL-C)], liver function markers [albumin (ALB), alanine aminotransferase (ALT), aspartate aminotransferase (AST), alkaline phosphatase (ALP), and gamma-glutamyl transferase (γ-GT)], uric acid (UA) and creatinine (Cr), were processed on a Siemens ADVIA2400 automatic biochemistry analyzer (Germany). Spot urine samples were sent to a central laboratory (Siemens Healthcare Diagnostics Inc, Tarrytown, New York, USA) to determine the urinary albumin/creatinine ratio (UACR). The estimated glomerular filtration rate (eGFR) was calculated using the Chronic Kidney Disease Epidemiology Collaboration (CKD-EPI) equation (8).

In accordance with Chinese adult obesity guidelines, BMI categories were defined as follows: underweight (BMI<18.5 kg/m^2^), normal weight (18.5≤ BMI <24 kg/m^2^), overweight (24≤ BMI <28 kg/m^2^), and obese (BMI ≥28 kg/m^2^) (9). In this study, based on their BMI, the patients were categorized into two main groups: the normal weight group (BMI <24 kg/m^2^) and the overweight/obese group (BMI ≥24 kg/m^2^). The homeostasis model assessment (HOMA) was employed to estimate insulin resistance (HOMA-IR) and β-cell function (HOMA-β). Specifically, HOMA-IR was calculated as [fasting insulin (μU/ml)×fasting glucose (mmol/l)]/22.5, while HOMA-β was calculated as [20×fasting insulin (μU/ml)]/[fasting glucose (mmol/l)-3.5] (10, 11). Insulin resistance was defined as HOMA-IR ≥2.8 (12). According to KDIGO guidelines, albuminuria was categorized into three stages (13) based on UACR: A1 (UACR <30 mg/g, normal), A2 (UACR 30–300 mg/g, moderately elevated), and A3 (UACR ≥300 mg/g, severely increased). Albuminuria stages A2 or A3 were classified as proteinuria. Proteinuria progression was defined as worsening from stage A1 to A2/A3 or from A2 to A3. Changes in BW (△BW), systolic blood pressure (SBP) (△SBP), diastolic blood pressure (DBP) (△DBP), HbA1c (△HbA1c) and UACR (△UACR) after COVID-19 infection were evaluated as absolute changes from pre-infection levels. According to WHO clinical criteria, COVID-19 severity was classified into non-severe, severe, and critical categories (14).

### 2.3 Statistical analysis

All data were analyzed using the Statistical Package for the Social Sciences (SPSS) software, version 26 (IBM Corp., Armonk, NY). Data were expressed as mean (standard deviation) or median (range) for continuous variables, and counts (%) for categorical variables. For laboratory results, we assessed whether the measurements were outside the normal range. Variables were compared between two groups using Student’s t-tests (for normally distributed continuous variables), Mann-Whitney U test (for nonnormally distributed continuous variables), and Pearson χ 2 test or Fisher exact test (for categorical variables). Multiple logistic regression analysis was conducted to investigate the association between body weight status and the progression of proteinuria after COVID-19 infection. Analyses for association were performed using BMI as categorical and continuous variables, respectively. *P* < 0.05 was considered statistically significant.

## 3 Results

### 3.1 Baseline characteristics of the patients infected with COVID-19 and comparison of indicators stratified by BMI (<24 kg/m^2^ and ≥24 kg/m^2^)

A total of 688 patients with T2DM were included in the study. The average age was 50 ± 12 years, 65.6% were male, and 58.7% had a history of hypertension. The median duration of diabetes was 3.6 (1.0, 8.7) years, with 40.1% having a duration of more than 5 years. At baseline, mean BW and BMI were 72.2 ± 13.7 kg and 25.41 ± 3.77 kg/m^2^, respectively. Mean HbA1c was 7.0 ± 1.6%. The median eGFR was 107.8 (94.63, 119.88) ml/min/1.73 m^2^. Additionally, 35% were treated with ACE-Inhibitors (ACEi) and/or angiotensin II receptor blockers (ARB), 58.4% were treated with sodium-glucose co-transporter-2 inhibitors (SGLT2i), and 35.6% were treated with glucagon-like peptide-1 receptor agonists (GLP-1 RA). Furthermore, 83.3% had received a COVID-19 vaccination (**Table 1**).

**Table 1:** Baseline demographics and clinical characteristics of the COVID-19 infected participants stratified by BMI.

Variable	Total (N = 688)	Normal weight (n=267, 38.8%)	Overweight/obese (n=421, 61.2%)	P value
Age (y)	50 ± 12	54 ± 11	48 ± 12	<0.001
Male (n/N, %)	451/688 (65.6%)	165/267 (61.8%)	286/421 (67.9%)	0.099
Smoking (n/N, %)	135/688 (19.6%)	52/267 (19.5%)	83/421(19.7%)	0.939
Hypertension (n/N, %)	404/688 (58.7%)	138/267 (51.7%)	266/421 (63.2%)	0.003
SBP (mmHg)	130 ± 17	126 ± 17	131 ± 16	<0.001
DBP (mmHg)	76 ± 10	73 ± 10	79 ± 10	<0.001
Diabetes duration (y)	3.6 (1.0, 8.7)	4.5 (1.3, 12.4)	3.4 (0.8, 7.8)	0.002
Duration >5years, (n/N, %)	273/681 (40.1%)	120/263 (45.6%)	153/418 (36.6%)	0.019
BW (kg)	72.2 ± 13.7	62.2 ± 7.7	78.5 ± 12.9	<0.001
BMI (kg/m^2^)	25.41 ± 3.77	22.13 ± 1.43	27.49 ± 3.30	<0.001
WC (cm)	91.32 ± 10.86	83.90 ± 7.03	95.82 ± 10.30	<0.001
HC (cm)	97.64 ± 8.20	94.54 ± 5.60	100.06 ± 6.68	<0.001
WHR	0.936 ± 0.067	0.906 ± 0.061	0.954 ± 0.064	<0.001
Visceral fat (cm^2^)	95 (67, 123)	69 (54, 90)	107 (82, 142)	<0.001
Subcutaneous fat (cm^2^)	173 (141, 220)	143 (108, 156)	203 (168, 251)	<0.001
FBG (mmol/L)	6.81 ± 2.16	6.65 ± 2.17	6.92 ± 2.15	0.11
HbA1c (%)	7.0 ± 1.6	7.0 ± 1.5	7.0 ± 1.7	0.702
HbA1c >7%, (n/N, %)	216/675 (32%)	77/261 (29.5%)	139/414 (33.6%)	0.269
HOMA-IR	2.76 (2.26, 3.37)	2.40 (2.07, 2.84)	2.96 (2.51, 3.71)	<0.001
HOMA-IR ≥2.8, (n/N, %)	303/632 (47.9%)	68/243 (28.0%)	235/389 (60.4%)	<0.001
HOMA-β	99.86 (81.28, 134.06)	88.86 (73.62, 110.66)	109.93 (86.47, 149.17)	<0.001
TC (mmol/L)	4.49 ± 1.19	4.45 ± 1.18	4.51 ± 1.19	0.572
TGs (mmol/L)	1.38 (0.98, 2.01)	1.14 (0.85, 1.69)	1.52 (1.10, 2.27)	<0.001
HDL-C (mmol/L)	1.10 ± 0.33	1.22 ± 0.35	1.03 ± 0.29	<0.001
LDL-C (mmol/L)	2.63 ± 0.99	2.57 ± 0.99	2.67 ± 0.98	0.184
ALT (U/L)	24 (17, 34)	21 (16, 29)	26 (19, 39)	<0.001
AST (U/L)	21 (18, 27)	20 (17, 24)	23 (18, 29)	<0.001
ALP (U/L)	69 (57, 83)	67 (55, 80)	70 (59, 84)	0.028
γ-GT (U/L)	22 (15, 37)	17 (13, 28)	26 (17, 43)	<0.001
ALB (g/L)	46 ± 3.7	45 ± 4	46 ± 4	0.002
BUN (mmol/L)	6.06 ± 2.17	6.25 ± 2.28	5.94 ± 2.10	0.072
Cr (µmol/L)	66 (55, 76)	66 (55, 77)	66 (54, 76)	0.58
UA (µmol/L)	315 (267, 377)	300 (253, 358)	330 (273, 387)	<0.001
eGFR (mL/min/1.73m^2^)	107.80 (94.63, 119.88)	104.59 (91.45, 115.91)	110.26 (96.82, 121.97)	<0.001
ACEi and/or ARB use (n/N, %)	241/688 (35.0%)	78/267 (29.2%)	163/421 (38.7%)	0.011
SGLT2i use (n/N, %)	402/688 (58.4%)	149/267 (55.8%)	253/421 (60.1%)	0.266
GLP-1 RA use (n/N, %)	245/688 (35.6%)	33/267 (12.4%)	212/421 (50.4%)	<0.001
COVID-19 vaccinated (n/N, %)	567/681 (83.3%)	213/262 (81.3%)	354/419 (84.5%)	0.278

Data are presented as the mean ± standard deviation (SD) or median (interquartile range) for continuous variables and proportion for categorical variables. BMI, body mass index; SBP, systolic blood pressure; DBP, diastolic blood pressure; BW, body weight; WC, waist circumference; HC, hip circumference; WHR, waist/hip ratio; FBG, fasting blood glucose; HbA1c, hemoglobin A1c; HOMA-IR, homeostatic model assessment of insulin resistance; TC, serum total cholesterol; TGs, triglycerides; HDL-C, high-density lipoprotein cholesterol; LDL-C, low-density lipoprotein cholesterol; HOMA-β, homeostasis model assessment of β-cell function; ALT, alanine aminotransferase; AST, aspartate aminotransferase; ALP, alkaline phosphatase; γ-GT, gamma-glutamyl transferase; ALB, albumin; BUN, blood urea nitrogen; Cr, creatinine; UA, uric acid; eGFR, estimated glomerular filtration rate; ACEi, ACE-Inhibitors; ARB, angiotensin II receptor blockers; SGLT2i, sodium-glucose co-transporter-2 inhibitors; GLP-1 RA, glucagon-like peptide-1 receptor agonists. Patients were classified into two groups: the normal weight group (BMI <24 kg/m^2^) and the overweight/obese group (BMI ≥24 kg/m^2^).

Of the 688 participants, 267 (38.8%) were classified into the normal weight group (BMI <24 kg/m^2^), which included 5 underweight individuals. The remaining 421 (61.2%) participants were categorized into the overweight/obese group (BMI ≥24 kg/m^2^), with 40.8% being overweight and 20.4% obese. Compared to the normal weight group, the overweight/obese group was younger and had a shorter duration of diabetes. This group also exhibited a higher prevalence of hypertension and elevated SBP and DBP. Furthermore, the overweight/obese group demonstrated significantly higher values for a broad range of anthropometric, body composition, and metabolic parameters, including BW, WC, HC, WHR, visceral fat, subcutaneous fat, HOMA-IR, HOMA-β, liver enzymes, UA, eGFR and TGs. Conversely, HDL-C was significantly lower in this group. In the overweight/obese group, there was a higher proportion of patients with HOMA-IR ≥2.8 and a greater utilization rate of ACEi and/or ARB, and GLP-1 RA. No significant differences were observed between the two groups for FBG, HbA1c, BUN, Cr, TC, LDL-C, the usage of SGLT2i and vaccination status (P >0.05) (**Table 1**).

### 3.2 UACR and proteinuria status by BMI group in COVID-19 infected cohort

Prior to COVID-19 infection, a total of 644 participants had UACR measurements. The median UACR was 12.79 (7.49-26.81) mg/g, and the proportion of proteinuria was 21.7% (17.8% in A2 stage and 3.9% in A3 stage). Participants in the overweight/obese group had a slightly higher baseline UACR [13.37 (8.06, 27.24) mg/g] compared to the normal weight group [11.42 (6.91, 26.53) mg/g, P = 0.042]. However, no significant difference was observed between the two groups in either the proportion of proteinuria (22.0% in normal weight group vs. 21.6% in overweight/obese group, P = 0.898) or its stages (**Table 2**).

**Table 2:** Comparison of UACR and proteinuria stages between normal weight and overweight/obese groups in the COVID-19 infected cohort.

Variable	Total (N = 644)	Normal weight (n=250)	Overweight/obese (n=394)	P value
UACR (mg/g)	12.79 (7.49, 26.81)	11.42 (6.91, 26.53)	13.37 (8.06, 27.24)	0.042
Proteinuria (n, %)	140 (21.7%)	55 (22.0%)	85 (21.6%)	0.898
A1(n, %)	504 (78.3%)	195 (78%)	309 (78.4%)	0.989
A2(n, %)	115 (17.8%)	45 (18%)	70 (17.8%)	
A3(n, %)	25 (3.9%)	10 (4%)	15 (3.8%)	

Data are presented as median (interquartile range) for continuous variables and proportion for categorical variables. UACR, urinary albumin/creatinine ratio. Patients were classified into two groups: the normal weight group (BMI <24 kg/m^2^) and the overweight/obese group (BMI ≥24 kg/m^2^). Albuminuria was categorized into three stages: A1 (UACR <30 mg/g, normal), A2 (UACR 30–300 mg/g, moderately elevated), and A3 (UACR ≥300 mg/g, severely increased) according to KDIGO guidelines.

### 3.3 Post-COVID-19 infection changes and medication utilization

Among the 688 participants with COVID-19 infection, 2.3% were diagnosed as severe cases, and no critical cases were diagnosed (**Table 3**). Changes in patients’ BW, BP or HbA1c were minimal within three months after COVID-19 infection. However, 8.5% of patients experienced proteinuria progression. Most patients remained stable (84.8%), with 6.7% showed regression (**Supplementary Table 1**). The incidence of proteinuria progression was significantly higher in the overweight/obese group (10.8%) compared to the normal weight group (4.9%) (P = 0.012) (**Table 3**). Recognizing that certain medication, such as SGLT2i, GLP-1 RA, ACEi and ARB, can reduce proteinuria, we also examined changes in their utilization within three months before or after COVID-19 infection. During this period, 22.8% of the participants initiated SGLT2i or GLP-1 RA, and 4.4% initiated ACEi/ARB, while 5.7% discontinued SGLT2i or GLP-1 RA, and 0.6% discontinued ACEi/ARB. Furthermore, a higher proportion of participants in the overweight/obese group (27.1%) initiated SGLT2i or GLP-1 RA compared to the normal weight group (16.1%) (P = 0.001). However, no significant differences were found in the proportion of participants discontinuing SGLT2i or GLP-1 RA, discontinuing ACEi/ARB or initiating ACEi/ARB between the two groups (**Table 3**).

**Table 3:** Post-COVID-19 infection changes and medication use by BMI category.

Variable	Total (N = 688)	Normal weight (n=267)	Overweight/obese (n=421)	P value
Severe COVID-19	16 (2.3%)	6 (2.2%)	11 (2.6%)	0.763
△BW (kg)	0.4 (-1.4, 2.0)	0.65 (-0.725, 2.3)	0.1 (-1.9, 1.9)	0.001
△SBP (mmHg)	3 (-7, 13)	4 (-5, 13)	4 (-9, 13)	0.266
△DBP (mmHg)	1 (-6, 7)	1 (-4, 6)	1 (-7, 7)	0.284
△HbA1c (%)	0.0 (-0.4, 0.3)	0.1 (-0.2, 0.3)	0 (-0.5, 0.3)	0.01
△UACR (mg/g)	0.02 (-5.85, 6.25)	-0.18 (-5.32, 5.33)	0.09 (-6.20, 6.87)	0.403
Proteinuria progression (n/N, %)	50/585 (8.5%)	11/225 (4.9%)	39/360 (10.8%)	0.012
SGLT2i or GLP-1 RA initiated ^*^ (n, %)	157 (22.8%)	43 (16.1%)	114 (27.1%)	0.001
SGLT2i or GLP-1 RA discontinued ^*^ (n, %)	39 (5.7%)	13 (4.9%)	26 (6.2%)	0.47
ACEi/ARB initiated ^*^ (n, %)	30 (4.4%)	12 (4.5%)	18 (4.3%)	0.891
ACEi/ARB discontinued ^*^ (n, %)	4 (0.6%)	1 (0.4%)	3 (0.7%)	0.57

Data are presented as the median (interquartile range) for continuous variables and proportion for categorical variables. *Indicates changes occurred within three months before and after COVID-19. BMI, body mass index; BW, body weight; SBP, systolic blood pressure; DBP, diastolic blood pressure; HbA1c, hemoglobin A1c; UACR, urinary albumin/creatinine ratio; SGLT2i, sodium-glucose co-transporter-2 inhibitors; GLP-1 RA, glucagon-like peptide-1 receptor agonists; ACEi, ACE-Inhibitors; ARB, angiotensin II receptor blockers.

### 3.4 Proteinuria progression in COVID-19 infected and uninfected patients

Subsequently, we compared the incidence of proteinuria progression between participants with and without COVID-19 infection. Baseline demographic and clinical data, including age, gender, SBP, diabetes duration, BMI, WC, HC, UACR and the proportion of proteinuria, were comparable between the 585 patients with COVID-19 infection and the 502 patients without COVID-19 infection (**Supplementary Table 2**). However, the incidence of proteinuria progression was significantly higher in participants with COVID-19 infection (8.5%) than in those without COVID-19 infection (5.4%, P = 0.042) (**Table 4**).

**Table 4:** Comparison of proteinuria progression in COVID-19 infected and uninfected patients.

Variable	Overall	Normal weight	Overweight/obese	P value
COVID-19 infection (n=585)	50/585 (8.5%)	11/225 (4.9%)	39/360 (10.8%)	0.012
No COVID-19 infection (n=502)	27/502(5.4%)	7/158 (4.4%)	20/344 (5.8%)	0.523
P value	0.042	0.835	0.016	

This significant difference was primarily attributable to the overweight/obese group among COVID-19 infected patients. Within the COVID-19 infected cohort, the incidence of proteinuria progression was significantly higher in the overweight/obese group (10.8%) compared to the normal weight group (4.9%, P = 0.012). Furthermore, this incidence in the infected overweight/obese group (10.8%) was also significantly higher than that in their uninfected overweight/obese counterparts (5.8%, P = 0.016) (**Table 4**). However, in the normal weight group, proteinuria progression was similar regardless of COVID-19 infection (4.9% vs 4.4%, P = 0.835). Additionally, among COVID-19 uninfected individuals, the incidence of proteinuria progression in overweight/obese patients (5.8%) was comparable to that in normal weight individuals (4.4%, P = 0.523) (**Table 4**).

### 3.5 Association of BMI and other factors with proteinuria progression after COVID-19 infection

To determine the association between body weight status and proteinuria progression after COVID-19 infection, logistic regression analyses was performed by adjusting for potential confounding factors, including age, sex, smoking status, hypertension, diabetes duration, eGFR, △HbA1c, and the initiation or use of specific medications (SGLT2i, GLP-1 RA, ACEi/ARB). The results revealed that patients with BMI ≥24 kg/m^2^ exhibited a 2.682-fold higher risk of proteinuria progression compared to those with BMI <24 kg/m^2^ (OR, 2.682; 95%CI, 1.273-5.648; P = 0.009) (**Table 5**). A significant correlation was also observed between BMI, as a continuous variable, and proteinuria progression, suggesting that every 1 kg/m^2^ increase in BMI was associated with a 13.1% increased risk of proteinuria progression (OR, 1.131; 95%CI, 1.039-1.23; P = 0.004, data not shown). Additionally, after COVID-19 infection, women had a 2.276 times higher risk of proteinuria progression than men (OR, 2.276; 95%CI, 1.123-4.610; P = 0.022). Furthermore, for every 1% increase in HbA1c in diabetic patients after COVID-19 infection, the risk of proteinuria progression increased by 62.8% (OR, 1.628; 95%CI, 1.189-2.228; P = 0.002) (**Table 5**).

**Table 5:** Risk factors for proteinuria progression after COVID-19 infection.

Factors	OR	95% CI	P value
Age (y)	1.014	0.98-1.048	0.425
Female	2.276	1.123-4.610	0.022
Smoking	0.413	0.118-1.445	0.166
Hypertension	0.798	0.334-1.909	0.613
Diabetes duration (y)	0.981	0.932-1.034	0.477
BMI ≥24 kg/m^2^	2.682	1.273-5.648	0.009
eGFR (mL/min/1.73m^2^)	1.004	0.986-1.021	0.696
△HbA1c (%)	1.628	1.189-2.228	0.002
Initiation of SGLT2i or GLP-1 RA	1.583	0.614-4.076	0.342
use of ACEi/ARB	0.738	0.337-1.618	0.448
use of SGLT2i/GLP-1 RA	0.962	0.468-1.981	0.917

Adjusted OR for the association between BMI and proteinuria progression in T2DM patients with COVID-19 infection. Multiple logistic regression analyses was adjusted for other confounding factors, including age, sex, smoking status, hypertension, diabetes duration, BMI, eGFR, △HbA1c, initiation of SGLT2i or GLP-1 RA, use of ACEi/ARB, use of SGLT2i/GLP1 RA. OR, Odds ratio; 95%CI, 95% confidence interval; BMI, body mass index; eGFR, estimated glomerular filtration rate; HbA1c, hemoglobin A1c; SGLT2i, sodium-glucose co-transporter-2 inhibitors; GLP-1 RA, glucagon-like peptide-1 receptor agonists; ACEi, ACE-Inhibitors; ARB, angiotensin II receptor blockers.

## 4 Discussion

In this retrospective study of patients with T2DM, we found that overweight/obesity significantly accelerates proteinuria progression following COVID-19 infection. The incidence of proteinuria progression in the infected overweight/obese group (10.8%) was significantly higher than that in the infected normal weight group (4.9%, P = 0.012). Notably, the incidence of progression in the infected overweight/obese group was also significantly higher than that in their uninfected overweight/obese counterparts (5.8%, P = 0.016). However, normal weight individuals showed similar incidence of progression regardless of their infection status (4.9% vs. 4.4%, P = 0.835). This distinct differential effect highlights a synergistic renal risk between COVID-19 infection and overweight/obesity in the T2DM population.

Proteinuria in COVID-19 patients can be attributed to the renal tropism of SARS-CoV-2. The virus directly infects renal tubular epithelial cells and podocytes via angiotensin-converting enzyme 2 (ACE2) binding, leading to acute tubular necrosis, collapsing glomerulopathy, the formation of protein reabsorption vacuoles, and protein leakage in Bowman’s capsule, all closely related to kidney injury (15, 16). Early studies consistently showed a high incidence of proteinuria with COVID-19 infection (17, 18). Given the known nephrotoxicity of the SARS-CoV-2, our cohort of COVID-19 infected patients with T2DM showed accelerated renal damage, with an overall incidence of proteinuria progression (8.5%) significantly higher than that of the non-infected, pre-pandemic control cohort (5.4%, P = 0.042). This result is consistent with findings from a cohort of 2212 individuals at post-COVID recovery clinics (PCRC) in British Columbia, Canada, which reported that patients with pre-existing diabetes at baseline exhibited a significantly higher risk of persistent proteinuria after COVID-19 (19).

However, this finding contrasts with result from the Hamburg City Health Study (HCHS). Their cross-sectional study demonstrated that individuals, a median of 9 months after non-severe COVID-19, had a similar occurrence of mildly increased UACR (4.5%) compared to non-COVID-19 controls (4.2%) (20), suggesting no ongoing kidney injury. This discrepancy may result from several differences in study design and cohort characteristics. Firstly, study populations varied significantly: our study focused on T2DM, a population more susceptible to renal complications, while the Hamburg study’s cohort consisted mainly of hypertensive individuals. Secondly, the dominant viral strains differed. The HCHS observed infections from early 2020 (wild-type, Alpha, Delta variants), while the infection in our study occurred in late 2022 to early 2023 (Omicron variants), which may have different pathogenicity and nephrotoxic effects. Lastly, follow-up duration plays a role: our observation period was limited to three months, which may have missed longer-term changes or recovery patterns. These variations underscore the need for continued research to fully understand COVID-19’s long-term renal consequences across diverse groups and viral types.

Our research further revealed that this progression was particularly pronounced in overweight/obese infected individuals. Our multiple logistic regression analysis confirmed that overweight/obesity was an independent and significant risk factor for proteinuria progression, associated with a 2.682-fold higher risk compared to those normal weight individuals after adjusting for key confounders (OR, 2.682; 95%CI, 1.273-5.648; P = 0.009). The dose-response relationship was further confirmed by the continuous analysis, suggesting that every 1 kg/m^2^ increase in BMI was associated with a 13.1% increased risk of proteinuria progression (OR, 1.131; 95% CI, 1.039-1.23; P = 0.004). Mechanistically, patients with diabesity—the deleterious intersection of type 2 diabetes and obesity—exhibit a pronounced susceptibility to the development and progression of albuminuria. These individuals endure a synergistic combination of chronic low-grade inflammation, lipotoxicity, glucotoxicity, and overactivation of the Renin-Angiotensin-Aldosterone System (RAAS). Collectively, these factors establish underlying endothelial dysfunction, glomerular hyperfiltration, and persistent podocyte injury. When these highly vulnerable patients contract COVID-19, the virus acts as a second hit to the kidneys. SARS-CoV-2 not only directly infects renal cells via ACE2 receptors but also triggers a systemic cytokine storm. As recently reported by Teng et al., viral infections such as COVID-19 can profoundly amplify the interaction of these pre-existing metabolic states, thereby exacerbating vascular endothelial damage and accelerating the progression of albuminuria (21). This dual-hit mechanism provides a compelling explanation for the high progression rate (10.8%) observed in our overweight/obesity patients with T2DM following COVID-19 infection.

Despite the overweight/obese group showing modest improvements in blood glucose control and weight, and having a significantly higher rate of SGLT2i or GLP-1 RA initiation compared to the normal weight group, their risk of proteinuria progression remained higher after COVID-19 infection. This suggests that the synergistic “dual-hit” of inflammatory and metabolic stress blunts the protective benefits of improved short-term metabolic control and renoprotective medication use. Therefore, with COVID-19 still prevalent, obesity intervention is crucial for the management of diabetes to prevent renal complications.

Interestingly, our study found that female sex was independently associated with an increased risk of proteinuria progression following COVID-19 infection (OR, 2.276; 95%CI, 1.123-4.610; P = 0.022). Consistent with our results, an observational study of 148 pediatric patients reported higher levels of proteinuria in females over a median 3-month follow-up after COVID-19 infection (22). Recent evidence also suggests that female sex is a key risk factor for long-term COVID-19 symptoms (23). These disparities may be related to sex-related differences in innate immunity, steroid hormones, and sex chromosome-related factors (24). The exact mechanisms underlying these sex-based differences in proteinuria progression after COVID-19 require further validation through larger-scale prospective and basic research studies.

Our study also revealed that every 1% increase in HbA1c led to a 62.8% increased risk of proteinuria progression in the infected patients. Although this finding aligns with previous studies demonstrating an independent correlation between HbA1c and elevated proteinuria (25), the short follow-up period of only three months in this study limits definitive conclusions about HbA1c and proteinuria progression.

Our study has several limitations. Firstly, it’s a single center, retrospective study with a small sample size, which may limit the generalizability of our findings. Secondly, we did not include critical cases of COVID-19, limiting our ability to analyze patients across various stages of the COVID-19 disease. A critical methodological point is our reliance on the pre-pandemic cohort as the primary control group. We acknowledge that a large contemporaneous non-infected cohort is ideal, but given that the available contemporaneous non-infected cohort had a limited sample size, the choice of the pre-pandemic cohort was necessary to ensure adequate statistical power for the key BMI subgroup analysis. Finally, our short follow-up period prevented us from assessing the long-term relationship between obesity and proteinuria progression after COVID-19 infection.

In conclusion, our study found that overweight/obese is an independent and significant driver of accelerated proteinuria progression in patients with T2DM post-COVID-19 infection. Therefore, obesity management plays a crucial role in preventing renal complications in T2DM patients after COVID-19 infection. We recommended that obese T2DM patients adopt appropriate weight loss strategies to better manage the risk of kidney damage following COVID-19 infection.

## Data Availability

The data analyzed in this study is subject to the following licenses/restrictions: The datasets generated during and analyzed during the current study are available from the corresponding author on reasonable request. Requests to access these datasets should be directed to Na Li, lena0113@126.com.
