# Supplementary material for: Overweight/obesity accelerates proteinuria progression in type 2 diabetes post-COVID-19: a comparison with a pre-pandemic uninfected cohort
Source: Front Endocrinol (Lausanne). 2026 Apr 17;17:1804593. doi: 10.3389/fendo.2026.1804593 (PMC13132767; doi:10.3389/fendo.2026.1804593)
Supplement: Supplementary file 1 [file Table1.docx]

**Supplementary Table 1.** Proteinuria outcomes post-COVID-19 by BMI category

|  | Total (N=585) | Normal weight (n=225) | Overweight/obese  (n=360) | P value |
| --- | --- | --- | --- | --- |
| Proteinuria outcome |  |  |  | 0.039 |
| Progression (n/N, %) | 50/585 (8.5%) | 11/225 (4.9%) | 39/360 (10.8%) |  |
| Stable (n/N, %) | 496/585 (84.8%) | 197/225 (87.6%) | 299/360 (83.1%) |  |
| Regression (n/N, %) | 39/585 (6.7%) | 17/225 (7.6%) | 22/360 (6.1%) |  |

**Supplementary Table 2.** Baseline demographics and clinical characteristics of the participants with and without COVID-19 infection

|  | COVID-19 uninfected (n=502) | COVID-19 infected (n=585) | P value |
| --- | --- | --- | --- |
| Age (y) | 50 ± 11 | 51 ± 12 | 0.473 |
| Male (n, %) | 356 (70.9%) | 389 (66.5%) | 0.118 |
| Hypertension (n, %) | 221 (44.0%) | 347 (59.3%) | <0.001 |
| SBP (mmHg) | 130 ± 16 | 130 ± 17 | 0.804 |
| DBP (mmHg) | 78 ± 10 | 77 ± 10 | 0.021 |
| Diabetes duration (y) | 2.8 (0.1, 8.8) | 3.0 (0.6, 8.1) | 0.088 |
| Duration >5years (n, %) | 194 (38.6%） | 226 (38.6%) | 0.997 |
| BMI (kg/m^2^) | 25.69 ± 3.63 | 25.40 ± 3.75 | 0.184 |
| WC (cm) | 91.50 ± 8.58 | 91.22 ± 10.47 | 0.717 |
| HC (cm) | 97.03 ± 6.39 | 97.19 ± 7.91 | 0.776 |
| FBG (mmol/L) | 8.49 ± 2.76 | 6.81 ± 2.20 | <0.001 |
| HbA1c (%) | 8.2 ± 2.0 | 7.0 ± 1.6 | <0.001 |
| UACR (mg/g) | 13.46 (7.17, 35.81) | 12.94 (7.52, 27.38) | 0.816 |
| Proteinuria (n, %) | 137 (27.3%) | 130 (22.2%) | 0.053 |

Data are presented as the mean ± standard deviation (SD) or median (interquartile range) for continuous variables and proportion for categorical variables. Abbreviations: SBP, systolic blood pressure; DBP, diastolic blood pressure; BW, body weight; BMI, body mass index; WC, waist circumference; HC, hip circumference; FBG, fasting blood glucose; HbA1c, hemoglobin A1c; UACR, urinary albumin/ creatinine ratio.
